# Age-associated sleep spindle characteristics in Duchenne muscular dystrophy

**DOI:** 10.1093/sleepadvances/zpae015

**Published:** 2024-03-14

**Authors:** Katharine C Simon, Chelsea Cadle, Neal Nakra, Marni C Nagel, Paola Malerba

**Affiliations:** Department of Pediatrics, School of Medicine, University of California, Irvine, Irvine, CA, USA; Pulmonology Department, Children’s Hospital of Orange County, Orange, CA, USA; Center for Biobehavioral Health, Abigail Wexner Research Institute, Nationwide Children’s Hospital, Columbus, OH, USA; Pulmonology Department, Children’s Hospital of Orange County, Orange, CA, USA; Department of Pediatric Psychology, Children’s Hospital of Orange County, Orange, CA, USA; Center for Biobehavioral Health, Abigail Wexner Research Institute, Nationwide Children’s Hospital, Columbus, OH, USA; Department of Pediatrics, School of Medicine, The Ohio State University, Columbus, OH, USA

**Keywords:** sleep spindles, development, Duchenne muscular dystrophy

## Abstract

Brain oscillations of non-rapid eye movement sleep, including slow oscillations (SO, 0.5–1.5 Hz) and spindles (10–16 Hz), mirror underlying brain maturation across development and are associated with cognition. Hence, age-associated emergence and changes in the electrophysiological properties of these rhythms can lend insight into cortical development, specifically in comparisons between pediatric populations and typically developing peers. We previously evaluated age-associated changes in SOs in male patients with Duchenne muscular dystrophy (DMD), finding a significant age-related decline between 4 and 18 years. While primarily a muscle disorder, male patients with DMD can also have sleep, cognitive, and cortical abnormalities, thought to be driven by altered dystrophin expression in the brain. In this follow-up study, we characterized the age-associated changes in sleep spindles. We found that age-dependent spindle characteristics in patients with DMD, including density, frequency, amplitude, and duration, were consistent with age-associated trends reported in the literature for typically developing controls. Combined with our prior finding of age-associated decline in SOs, our results suggest that SOs, but not spindles, are a candidate intervention target to enhance sleep in patients with DMD.

**Statement of Significance**

Research characterizing age-associated changes in sleep events is scarce but needed in rare pediatric clinical populations. Our prior work identified an age-associated decline in slow oscillations in patients with Duchenne muscular dystrophy. Here we characterize typically developing sleep spindles in this same population. Together this research points to specific targetable sleep electrophysiological events for future intervention to benefit sleep and cognition.

## Introduction

The first two decades of life are a significant time of cortical development that corresponds with the emergence of higher-level cognitive abilities [1]. The underlying neural maturation supporting this cognitive development is mirrored by changes in sleeping brain rhythms across the first two decades of life [2]. Noninvasive insight into the maturation of neural networks can be gleaned from the intrinsic electrophysiological properties of sleeping brain rhythms [2, 3]. Slow wave activity (SWA, power in the 0.1–4 Hz range) and sleep spindles (waxing-and-waning bursts of coordinated spiking in the 10–16 Hz range) are the signature brain rhythms that characterize non-rapid eye movement (NREM) sleep. Properties of both SWA and spindles are known to mirror typical brain maturation [3–9]. The SWA peak shifts across the posterior-to-anterior axis from childhood to adolescence, paralleling brain changes in gray matter volume, cortical thickness, and skill maturation [2–4, 6, 10–12]. Similarly, in typically developing children, sleep spindles show topographical and morphological shifts, with greatest increases in frequency and density observed between childhood and adolescence [13–16]. Leveraging these typical maturation shifts in sleep oscillation electrophysiology can elevate sleep features as informative biomarkers of pathological development.

Duchenne muscular dystrophy (DMD) is a degenerative neuromuscular disease that primarily affects males and is caused by reduced or absent dystrophin [17–23]. As DMD progresses, cardiorespiratory functions become compromised, resulting in prominent sleep disturbances. Sleep research in patients with DMD has primarily focused on the consequences of respiratory decline in sleep quality and quantity, but has largely overlooked the changes in sleeping brain oscillations [24–29]. Patients with DMD also present with cognitive impairment, thought to be related to altered dystrophin expression in the brain, including in the hippocampus [30, 31], a region crucial to the acquisition and retention of episodic memories [32, 33]. Approximately half of patients with DMD show cognitive delays [34, 35], with memory identified as a specific deficit [20, 22, 31, 36–41]. Since sleep facilitates the stabilization of newly formed hippocampal-dependent memories [42, 43], and improved memory performance after sleep has been causally linked to slow oscillations (SOs, 0.5–1.5Hz, large coordinated waves that contribute to SWA) [44–49], spindles [50], and SO–spindle coordination [51, 52], a full characterization of the sleep brain dynamics of patients with DMD can contribute to understand (1) the relation between sleep changes and disease progression and (2) the physiological substrate of the sleep and cognition deficit in this clinical population.

In our recent work, we characterized the changes in SOs between childhood and late adolescence in this population and found an age-associated decline in SO density and amplitude [53]. This reduction paralleled DMD disease progression, implicating specific time points in which sleep-based interventions might be beneficial. Whether sleep spindles show similar DMD disease-affected developmental changes in topography or morphology is unknown, and insights in their age-associated maturation could have important implications for the cognitive delays present in some patients. In this study, we retrospectively evaluated the same DMD patient’s clinical sleep studies as in Simon et al. (2021). We detected spindles in our dataset and characterized the changes between NREM sleep stages and age in spindle duration, amplitude, frequency, and density. Given that SOs are affected by DMD in an age-dependent way, we anticipated that spindle properties would show age-associated trends different than those found in the literature for typically developing children and adolescents.

## Methods

### Participants

The retrospective clinical sleep study records of 27 male pediatric patients (age M = 12.93, SD = 4.7, range = 4–20) with DMD (*n* = 24) and Becker muscular dystrophy (*n* = 3) were analyzed to characterize age-associated changes in sleep spindles. Only seven youths in total walked independently at the time of polysomnography, while the rest were dependent upon wheelchairs. Within our patients, six were noted to have a cognitive delay, with no further details provided as to the extent. Twenty-one youths were prescribed a variety of medications at the time of their sleep study. Common medication classes prescribed included glucocorticoids, angiotensin-converting enzyme inhibitors, beta-blockers, bronchodilators, and gastroesophageal reflux treatments. Institutional review board approval to evaluate the medical records and sleep studies was jointly received from the Children’s Hospital of Orange County (CHOC) and the University of California, Irvine.

### Records

All records were conducted between 2015 and 2018 at CHOC. The sleep studies were clinically indicated per standard medical care guidelines. Our prior research, Simon et al., 2021, evaluated these records for SOs in relation to three age groups (child, early adolescent, and late adolescent). For the current study, we maintained the same age groups, splitting the youth into children (age range = 4–9; M = 7.4 years, SD = 1.9, *n* = 9), early adolescent (age range = 10–15 years; M = 12.6, SD = 1.8, *n* = 8), and late adolescent (age range = 16–20 years; M = 17.6, SD = 1.7, *n* = 10). More details on the clinical sleep studies can be found in Simon et al. (2021). Briefly, we obtained 28 total records, of which 21 were baseline sleep studies to assess sleep-disordered breathing. Five additional sleep studies obtained were conducted as full-night positive airway pressure (PAP) titration sleep studies. The last two sleep studies obtained were split during the night, initially monitoring for obstructive sleep apnea (OSA) and then treated with PAP. We removed these two split studies from the final analyses due to the sleep fragmentation that results from PAP administration. We retained the titration studies as a clinical intervention to titrate the PAP devices that occur outside of the monitoring room, minimizing arousal and sleep fragmentation of the patient. For general sleep apnea indices, please see Simon et al. (2021). No differences in apnea events were present across youth. Additionally, one patient’s record was removed due to extensive artifact, one was removed due to spindles detected 3+ SDs above the mean at all electrode sites, one subject did not have occipital electrodes in the sleep study and was excluded from regional analyses, and lastly, one youth’s C3 electrode was removed from analysis due to malfunction. In total, we had 24 subjects in the final sample, with 23 used for all regional-based analyses.

### Sleep recording and scoring

The sleep studies occurred in a hospital-based setting and were administered by registered polysomnography technicians according to the American Academy of Sleep Medicine (AASM) guidelines between 2014 and 2018 at CHOC [54]. Polysomnography (PSG) records included electroencephalogram (EEG), electrocardiogram, electromyogram (EMG), electrooculogram (EOG), oronasal airflow, nasal pressure, PAP pressure (if indicated per patient, see above for details), oximetry, end-tidal or transcutaneous CO_2_, thoracoabdominal belts, snoring microphone, and video. PSG was collected using Neurofax EEG-1200 system (Nihon Kohden, Tokyo, Japan) and scored on Polysmith version 11. Registered polysomnography technicians applied the PSG according to the 10–20 placement with the EEG montage including six electrodes (F3, F4, C3, C4, O1, and O2), EOG, EMG, and mastoid (A1 and A2) [55]. All electrodes were contralaterally referenced to the mastoids. Recordings were sampled at 200 Hz and, for all EEG/EOG channels, a high-pass filter was set at 0.3 Hz and a low-pass filter at 35 Hz. For the thoracoabdominal belts, a high-pass filter was set at 0.1 Hz and a low-pass filter was set at 15 Hz. Registered polysomnographic technologists scored the recordings in 30-second epochs following standard AASM scoring criteria for stages N1, N2, N3, and REM [54]. All scored records were confirmed by a board-certified pediatric sleep physician. General sleep scoring (total sleep time [TST], sleep efficiency [SE], and minutes in each stage [N1, N2, N3, and REM]) can be seen in Simon et al. [53].

### Spindle detection algorithm

We applied a spindle detection algorithm previously used by Malerba et al. (2022), in-house coded with Matlab (TheMathWorks), which builds on spindle properties including frequency [56], waxing–waning profile, and sigma prominence in a time window of a detected spindle [57]. We analyzed each EEG channel separately for spindles in stages scored as N2 or N3. First, data were filtered between 9 and 16 Hz (Butterworth, band-pass filter of order 6). Its root mean square on 100-ms-long epochs was z-scored on 30-second-long epochs. Peaks of these z-scored epochs with a minimal prominence of 0.5 and minimal height of 3.5 (consistent with [58, 59]) were detected as a candidate spindle. For each candidate spindle, we isolated the time interval 1 second before and 1 second after the identified peak and computed the Morlet Wavelet spectrogram with the cwt function in Matlab. The spectrogram was used to identify individual spindle start times, end times, amplitude, and frequency. Our algorithm for spindle detection is consistent with previously reported algorithms [56, 57, 59–61]. The algorithm is introduced in detail in Malerba et al. [62].

Spindle characteristic measurements characterized were density, frequency, amplitude, and duration. Across the night, at each electrode, we measured spindle density which we defined as the count of all spindles detected at a specific sleep stage (N2 or N3) divided by the total time (seconds) spent in that specific sleep stage, thus accounting for the larger presence of N3 than N2 in the sleep night (see Simon et al., 2021). To identify the frequency of the spindles using the spectrogram in 2-second-long time intervals around the spindle peak, the level of maximum power in the sigma range was identified, marking an ellipse shape in time and frequency, with the spindle frequency as the value at the symmetric center of the ellipse. Spindle duration (in seconds) is the time between the start and end time of the detected spindle. Spindle amplitude was identified as the largest voltage in the detected spindle event in the filtered 9 and 16 Hz data using an order 6 Butterworth band pass.

### Spindle—Slow Wave Activity coupling

Coupling between spindle events and activity in the slow frequency range (0.5–4 Hz) was quantified by establishing SWA phase of each spindle event, and then computing their circular mean to find a phase-locking length and the locking phase. Calculations were conducted for events in N2 and N3 separately. For a given detection electrode, in order to establish SWA phase for each spindle event, we first had to isolate epochs of sleep that had enough SWA power that the phase in this frequency range could be considered a well-defined object (rather than effectively filtered noise). Hence, we first calculated the SWA power in 5-second-long windows with fast Fourier transforms (“fft” function in Matlab) and summed the area under the curve for time-bin-based power spectra in the SWA range. We then determine the median SWA power across all 5-second-long epochs of both given sleep stages (N2 and N3). Five-second-long intervals in which SWA power exceeded 1.5× the median for N2 + N3 sleep were considered times in which SWA phase could be extracted reliably. Then, for each spindle event detected at the same electrode, we asked if the spindle occurred at a time (referred to the peak time of spindle amplitude, as identified by our spindle detection algorithm) of reliable SWA activity epochs, and if so, we extracted the SWA phase of the spindle time. By computing the circular mean of the complex values corresponding to all the spindle-time SWA phases identified, we established a phase-locking length for each electrode and each sleep stage in every participant. The percentage of spindles that did occur at times of reliable SWA power was calculated as the percentage of “coupled spindles.” The resulting vector length can be interpreted as an index ranging from 0 (where the spindles equally co-occurred across the entire range of SWA phases) to 1 (where all spindles happened at the exact same phase).

Of note, our previous study did not focus on SWA but rather on SOs. Since our previous analysis showed an age-associated deficit in SOs, driven by density and amplitude of single events, it was not appropriate to conduct a locking analysis based on SO events, as it would have introduced a bias in our group comparisons. Instead, we choose to analyze how spindles coordinate with SWA. Compared to SO event detection, SWA is a measure less sensitive to universal thresholds and more attuned to an individual’s organization of EEG power across bands.

### Statistical analyses

To evaluate spindle characteristics across the entire group, we first conducted one-way repeated measure ANOVAs with stage (N2 and N3) and region (frontal, central, and occipital) for sleep spindle density, frequency, and amplitude. For each region, we averaged the two associated electrodes (e.g. F3 and F4 were averaged for the frontal region [M_Front_], C3 and C4 were averaged for the central region [M_Cent_], and O1 and O2 were averaged for the occipital region [M_Occ_]). To evaluate the relationship between N2 and N3 for all spindle characteristics, we conducted Pearson *r* correlations at the C3 electrode. To assess age-associated changes in spindle characteristics within each sleep stage (N2 and N3), we conducted one-way ANOVAs with between-subjects factor of age (child, early adolescent, and late adolescent) for dependent variables of spindle density, amplitude, and frequency. For each sleep stage, we also separately conducted two-way repeated measures ANOVAs with between-subjects factor of age (child, early adolescent, and late adolescent) and within-subject factor of region (frontal, central, and occipital) for dependent variables of spindle density, amplitude, duration, and frequency. For all post hoc *t*-tests, we applied Bonferroni correction to account for multiple comparisons and report postcorrection *p*-values. We further used the Greenhouse–Geisser correction method to correct the degrees of freedom when a violation in sphericity was indicated by Mauchly’s test [63, 64].

## Results

### Sleep characteristics

All sleep characteristics were previously reported in Simon et al. (2021) and briefly summarized here. For all statistical analyses, please refer to Simon et al. (2021). Across the age groups, children, early adolescents, and late adolescents spent similar time in N1, N2, and REM, but significantly different amounts of time in N3. Those in the child group spent the longest amount of time in N3, compared to early adolescents, and late adolescents. Given the nature of the clinical sleep studies being performed, all participants spent less time asleep than what is typically reported for normative values of TST across this age range. Lastly, no significant differences were detected in respiratory events (i.e., apneas) across the groups.

### Spindle characteristics for sleep stages N2 and N3

In our retrospective dataset of sleep studies in children with DMD, we algorithmically detected spindles in sleep stages N2 and N3 at each recording electrode, and averaged spindle properties in the frontal, central, and occipital regions. We were interested in evaluating spindle density, amplitude, frequency, and duration, separately in N2 and N3. Details on dataset, sleep characteristics, and spindle detection are reported in Methods and Simon et al. (2021). We ran a repeated measures ANOVA of stage (N2 and N3) by region (Fs, Cs, Os) separately for spindle density, frequency, duration, and amplitude.

**Figure 1.**
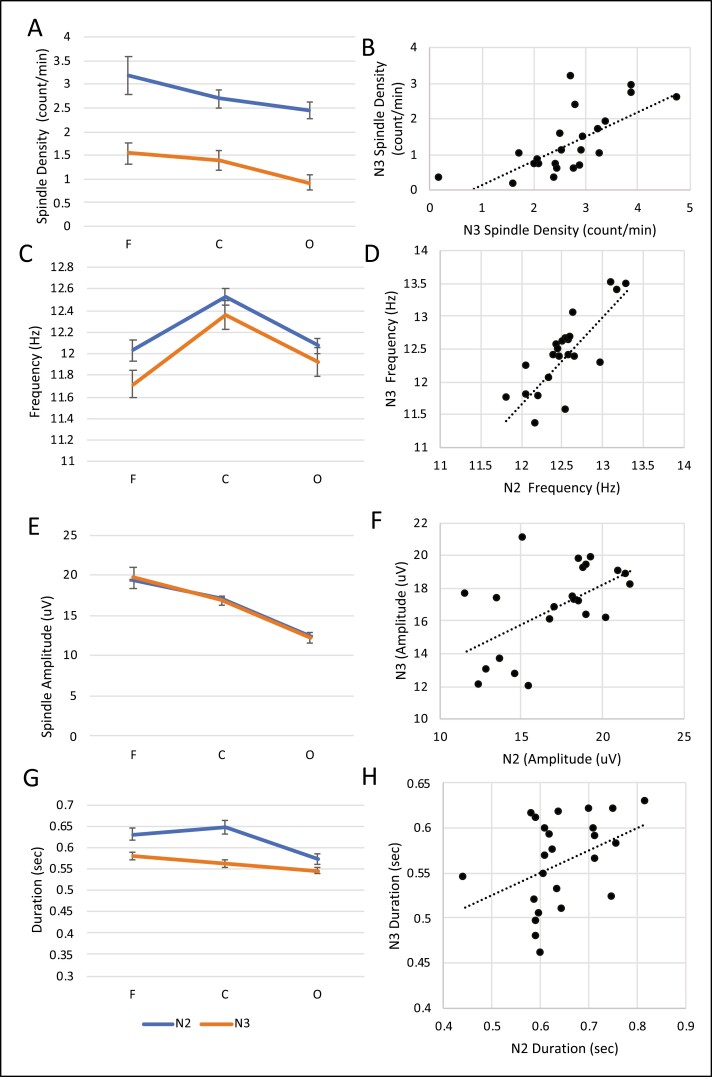
Spindle differences by region and sleep stage, N2 or N3. Each region is comprised of two electrodes, e.g. F region is an average of values in F3 and F4. (A) Spindle density was significantly greater in N2 than N3, and significantly greater at each anterior compared to posterior site. (B) A trending correlation between N2 and N3 for spindle density was observed. (C) We found significantly higher spindle frequency in N2 than N3, and at central than frontal or occipital electrode sites. (D) A significant correlation between N2 and N3 in spindle frequency was observed. (E) We found no significant difference in amplitudes between sleep stages but did find that amplitude was significantly higher in anterior compared to posterior sites across the brain. (F) A significant correlation between N2 and N3 in spindle amplitude was observed. (G) Significant differences were observed between N2 and N3 in spindle duration, with the longest duration present in the frontal cortex and the shortest at the occipital cortex. (H) There was no significant correlation between duration in N2 and N3. All right-sided correlation figures show detected spindles at C3 electrode in both N2 and N3 stages.

For spindle density (Figure 1A), measured as the total count/seconds of sleep within the stage, we found a main effect of stage (F(1, 22) = 106.955, *p* < .001, η_p_^2^ = 0.829) and region (F(1.450, 31.901) = 11.627, *p* < .001, η_p_^2^ = 0.346), and did not find an interaction between stage and region (*p* = .486, *n*^2^ = 0.032). Overall subjects had greater spindle density in N2 than N3 (mean difference = 1.429, SE = 0.138), and significantly greater spindle density at the frontal (M = 2.132, SE = 0.176) and central regions (M = 1.991, SE = 0.173) than the occipital region (M = 1.68, SE = 0.152). We found significant correlations within regions between N2 and N3; at the frontal regions (*r* = 0.631, *p* < .001), central regions (*r* = 0.629, *p* < .001), and occipital regions (*r* = 0.568, *p* = .005, Figure 1B).

For spindle frequency (Figure 1C), we found a main effect of stage (F(1, 22) = 9.055, *p* = .006, η_p_^2^ = 0.292) and region (F(2, 44) = 26.247, *p* < .001, η_p_^2^ = 0.544), but no interaction between stage and region (*p* = .398). Overall, spindle frequency was higher in N2 (M = 12.2, SE = 0.069) than N3 (M = 12.02, SE = 0.114). We found higher frequency spindles at the central region (M = 12.43, SE = 0.1) compared with frontal (M = 11.872, SE = 0.111) and occipital (M = 11.997, SE = 0.089; *p* < .001), but no significant difference between frontal and occipital regions (*p* = .190). We found significant correlations within regions between N2 and N3; at the frontal regions (*r* = 0.746, *p* < .001), central regions (*r* = 0.729, *p* < .001), and occipital regions (*r* = 0.542, *p* = .008, Figure 1D).

For spindle amplitude (Figure 1E), we found a main effect of region (F(1.354, 29.78) = 46.086, *p* < .001, η_p_^2^ = 0.677) but no main effect of stage (*p* = .920) nor interaction of stage by region (*p* = .248). Amplitude was significantly higher at the frontal region (M = 19.513, SE = 1.21), followed by the central region (M = 16.476, SE = 0.762), and lastly, the occipital region (M = 12.3, SE = 0.608, *p* < .001). We found significant correlations within regions between N2 and N3; at the frontal regions (*r* = 0.876, *p* < .001), central regions (*r* = 0.758, *p* < .001), and occipital regions (*r* = 0.666, *p* < .001, Figure 1F).

For spindle duration (Figure 1G), we found significant main effects of stage (F(1, 22) = 17.945, *p* < .001, η_p_^2^ = 0.449), region (F(2, 44) = 17.366, *p* < .001, η_p_^2^ = 0.441), and an interaction of stage by region (F(2, 44) = 12.771, *p* < .001, η_p_^2^ = 0.367). Duration was longer in N2 (M = 0.617, SE = 0.013) than N3 (M = 0.564, SE = 0.007). Duration also differed significantly between frontal (M = 0.605, SE = 0.009) and occipital (M = 0.560, SE = 0.008) regions, and between central (M = 0.608, SE = 0.011) and occipital (*p* < .001) regions. There was no significant difference between frontal and central regions (*p* = .807). Within each region, spindles were significantly longer in duration during N2 than N3 (*p* < .02). Further, within N2, spindle duration at frontal and central regions were significantly longer than at the occipital region (*p* < .001) regions, but not between each other (*p* = .089). For N3, spindle duration was significantly longer between frontal and occipital regions (*p* < .001) and between central and occipital regions (*p* = .05). We did not find a significant correlation between N2 and N3 within frontal regions (*p* = .261) or central regions (*p* = .052) or occipital region (*p* = .028 which did not withstand Bonferroni correction, Figure 1H).

### Age-associated differences in sleep spindle characteristics

**Figure 2.**
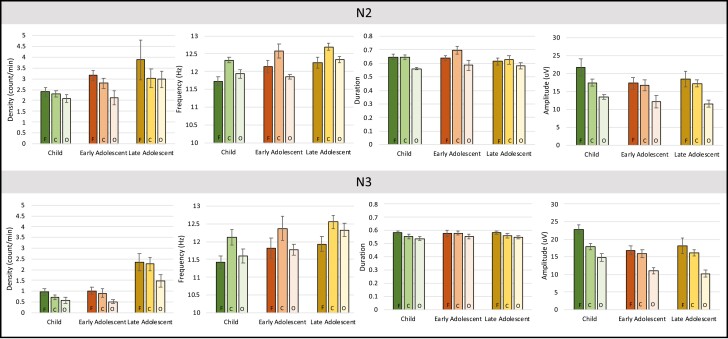
Sleep spindle characteristics by age and sleep stage. Age-associated changes in (left to right) spindle density (count/second), frequency (Hz), amplitude (uV), and duration (seconds) were found in N2 (top panel) and N3 (bottom panel). For each age group (childhood, early adolescence, and late adolescence), we show the regional differences in frontal electrodes (left side, darkest bars), central electrodes (middle bar, middle hue), and occipital electrodes (lightest bar, right side).

We also evaluated if age influenced the spindle characteristics (Figure 2). For spindle density, we found a main effect for stage (F(1, 20) = 126.649, *p* < .001, η_p_^2^ = 0.864) and region (F(1.426, 28.513) = 13.056, *p* < .001, η_p_^2^ = 0.395), and an interaction of stage by region by age group (F(3.008, 30.076) = 3.309, *p* = .033 η_p_^2^ = 0.249). We did not find any other significant interactions (*p* > 0.083). We also had a significant between-group effect (F(2, 20) = 5.204 *p* = .015 η_p_^2^ = 0.342; (M_Child_ = 1.5, SE = 0.21; M_EA_ = 1.75, SE = 0.28; M_LA_ = 2.465, SE = 0.21)), with oldest subjects having the highest rates of spindles compared to youngest (*p* = .005) and a trending difference with early adolescents (*p* = .063) but no significant difference between child and early adolescent (*p* = .36). Per the three-way interaction, for all groups, at each region, there were greater spindles in N2 than N3 (*p* < .001), which is a confirmatory finding of the nature of N2 compared to N3. When evaluating within age group, for children, we found no spindle differences across regions after correcting for Bonferroni within either N2 (*p* > 0.165) or N3 (*p* > 0.605). In contrast, for early adolescents, we found a significant difference within N2 between frontal and central with occipital regions (Fs–Os *p* < .001; Cs–Os *p* < .001) but not between frontal and central regions (*p* = .221). For late adolescents, we found no significant difference between regions for N2 (*p* > 1.0) but found a significant difference in N3 between central and occipital ranges (*p* = .005) and a trending effect between frontal and occipital (*p* = .08).

For spindle frequency, we found a significant main effect of stage (F(1, 20) = 8.165, *p* = .01, η_p_^2^ = 0.29) and region (F(2, 40) = 25.281, *p* < .001; η_p_^2^ = 0.558) but no other interactions (*p* > 0.323). We did find an age-associated difference (F(2, 20) = 3.869, *p* = .038) with a significant difference in frequency between children (M = 11.856, SE = 0.126) and late adolescents (M = 12.352, SE = 0.126; *p* = .035) but no other significant differences with early adolescents (M = 12.09, SE = 0.169; *p* > 0.685).

In spindle amplitude, we found an effect of region (F(1.337, 26.744) = 39.784, *p* < .001, η_p_^2^ = 0.665). We found no other significant main effects, interactions, or group differences (*p* > 0.142). We found significant amplitude differences anteriorly to posteriorly. Frontal regions had the highest amplitude (M = 19.151, SE = 1.21), followed by central regions (M = 16.446, SE = 0.772), followed by occipital regions (M = 12.209, SE = 0.57).

For spindle duration, we found significant main effects for stage (M(1,20) = 17.21, *p* < .001, η_p_^2^ = 0.463), region (F(2, 40) = 17.89, *p* < .001, η_p_^2^ = 0.472, and an interaction of stage by region (F(2, 40) = 15.07, *p* < .001, n^2^ = 0.43). We did not find an age-based difference (*p* = .687) nor other interactions (*p* > 0.139).

### Spindle—slow wave coupling

For all detected spindles, we evaluated how many co-occurred with SWA (0.5–4 Hz), focusing on how much SWA power was found at the time of spindle peak compared to the median amount of SWA power in N2 and N3 across the night (see Methods). If a spindle happened at a time when SWA power exceeded 1.5× the median, we labeled that spindle “coupled” to SWA. We found a significant effect of stage on fraction of spindles coupled to SWA (F(1, 22) = 80.286, *p* < .001, η_p_^2^ = 0.785). Post hoc tests revealed significantly higher spindle coupling to SWA in N3 than N2 (N2 M = 0.107, SE = 0.019; N3 M = 0.596, SE = 0.471), likely due to the higher density of SWA power characteristic of the sleep stage. We also found a trending effect of region (F(1, 22) = 3.406, *p* = .706, η_p_^2^ = 0.134) but did not find an interaction between spindle and region (*p* = .259, η_p_^2^ =0.059). We then conducted the repeated measures ANOVA with age group but found no age-associated coupling differences or interactions (*p* > 0.332).

Once we had established how many spindles were coupled to SWA, we looked to quantify if the occurrence of spindles during high SWA power was based on locking of sleep spindles to specific SWA phases. To that end, we analyzed the vector length resulting from circular averaging of SWA phases of all coupled spindles (“coupling strength”). We found a significant effect of sleep stage on coupling strength (F(2, 22) = 4.381, *p* = .048, η_p_^2^ = 0.166), with higher coupling strength during N2 than N3. We found no other differences in coupling strength, nor did we find any differences in coupling strength across our age groups (*p* > 0.083).

We found no significant difference in locking phase in either stage or region (*p*>0.361). In our age group analyses, we did find a significant interaction of stage by group (F (2, 20) = 4.066, *p* =0.033, η_p_^2^ =0.289). Post hoc analyses revealed that early adolescents’ spindles locked to SWA at phases that differed between N2 and N3. During N2, the preferred phase was 0.19, corresponding to the depolarized portion of SWA (akin to an upstate for SOs); in contrast, the preferred coupling SWA phase for spindles in N3 was −1.60, similar to the half point in the transition to upstate in an SO. We found no other significant locking phase differences in children or late adolescents (*p* > 0.4). We also found a significant age group interaction with region for preferred locking phase (F (2, 20) = 3.489, *p* = .05, η_p_^2^ = 0.259). Post hoc tests revealed a significant difference in the locking SWA phase for spindle occurrence in late adolescents between the frontal and central regions, with frontal regions coupling at −0.21 (again in depolarized activity) and central regions coupling at −1.36, (i.e. the activity following the trough about halfway to complete depolarization, corresponding to a transition to upstate in an SO).

## Discussion

We detected and characterized sleep spindles in overnight clinical sleep records from pediatric patients with DMD/BMD. Our current study builds upon our prior work that characterized this population’s age-associated decline in SOs (Simon et al., 2021). Here, we focused on age-associated sleep spindle changes in density, frequency, amplitude, and duration. Our results comparing spindle characteristics in N2 to N3 are in line with the existing literature in healthy adolescents. We found that spindles during N2 were higher in density, higher in frequency, and longer in duration. Spindle density regional differences were also present, with the highest rate in the frontal region and the lowest rate in the occipital region. In the central region, higher frequency spindles were observed compared to frontal or occipital regions. Of note, this increase in frequency from frontal to central locations is consistent with literature observing a similar topographical organization of spindle frequency across development [16]. In evaluating age-associated changes in spindle characteristics, we found that spindle density and frequency increased with age, while no age-associated differences in spindle amplitude or duration were detected. Age-associated regional spindle density differences were present in early adolescents, but not in children or late adolescents.

The detected spindles and their characteristics in our DMD/BMD sample are largely consistent with the existing spindle literature in healthy individuals [13–15, 62]. These initial results suggest that the thalamocortical spindles might not be directly affected by dystrophin loss experienced by patients with DMD/BMD. This is in line with research noting that dystrophin is not expressed in the thalamus [65, 66]. Dystrophin isotopes in the brain do appear to be concentrated in the hippocampus, cortex, and cerebellum [30, 38]. The heterogeneity of Xp21 genetic mutations causing the disorder results in a spectrum of decreased dystrophin expression and cognitive delays [31]. In the brain, alterations in synaptic terminal integrity, plasticity, and regional cellular signal integration have been identified [17, 22, 65, 67, 68]. Postmortem assessments in patients with DMD have also revealed brain degeneration at both the structural and cellular levels [30, 69–74]. In line with the heterogeneity of DMD expression in patient’s subcortical areas, a postmortem study of healthy adults showed that dystrophin isoform expression was highest in the hippocampus and amygdala, and also found high expression in the lateral nucleus, which is connected to the thalamus. Higher expression was also found in the subthalamus [38]. Overall, the limited papers discussing the thalamus in DMD sequelae suggest a role for altered cerebellar-thalamocortical projections underlie cognitive delays [75], rather than a directly thalamic influence of DMD. Despite these disease-associated neural consequences, the general characteristics of spindles we observed are within the normal limits in comparison with existing literature. Future research expanding on our analysis of spindle features in DMD could explore if subtle properties more directly linked to thalamic-cortical coordination, like coordination of spindles across the electrode manifold, are affected in DMD.

When evaluating age-associated changes in spindles, we found that children with DMD/BMD also had spindle characteristics generally consistent with prior developmental research [14, 15, 76, 77]. Two recent large scale studies by Kwon et al. [14] and Purcell et al. [15] have characterized normative spindle characteristics across development. Kwon et al. identified normative values for spindle characteristics in healthy 567 youth between 0 and 18 years using polysomnography from 19 channels. Purcell et al. evaluated spindles at 2 central electrodes in over 11 000 individuals across the lifespan, with a subset of subjects between 4 and 18 years. In line with both their developmental findings, we, too, found that spindle density significantly increased with age. However, we observed that rates significantly differed between our youngest and eldest age groups, but not between closer age groups (i.e., children and early adolescents). This rate change is slightly different than what was observed by Kwon et al., who found a linear increase in spindle rate between age 3 and 14 years, but a stable spindle rate in later adolescence. Our age-related rate difference could suggest spindles may be slower to increase in patients with DMD, or alternatively, could be influenced by our small sample size reducing our ability to detect rate variation in smaller age ranges. We also found significant N2 spindle density differences between each region in the early adolescent group, with the greatest in the frontal, then central, and then occipital regions. This frontal distribution predominance was also found by Kwon et al. in similar age ranges. In late adolescents, we observed significantly higher rates of spindles in the central compared to occipital regions. This morphological pattern was also observed by Kwon et al. However, we found no regional differences in spindle rate in our child group. This contradicted work by Kwon et al. who found that spindle distribution was dominant in the frontal regions from age 5 to 10 years. These age-associated regional differences may be linked with underlying brain maturation. Kurth et al. (2010) demonstrated that the changing SWA maximal peak across development mirrored underlying brain maturation. They also found topological patterns in sigma, demonstrating maximal sigma peak in frontal and central regions in late adolescents, but only a frontal maximal peak in earlier childhood. We show similar frontal/central regional density increases in our early and late adolescent groups but no regional differences in our child group. Our observation of increased spindle frequency in late adolescence compared to childhood also paralleled Kwon et al.’s findings. Interestingly, we observed no age-associated differences in spindle amplitude or duration. Kwon et al. found a small, but not always statistically significant, increase in duration when comparing toddler years and late adolescence. Our small sample size at each age range likely influences our ability to detect this small, nuanced change. Altogether, our findings further add evidence that the emergence and development of thalamocortical spindles in our patient population might not be directly affected by DMD disease progression.

One facet of DMD/BMD that is less discussed is the cognitive deficits, with memory being one of the selective areas of impairment [36, 40]. The cognitive deficits and comorbid neurobehavioral disorders present are heterogenous in nature, likely paralleling the underlying genetic mutations causing the disorder [35, 78, 79]. Sleep is known to be a brain state that is optimal for memory, with SOs, thalamocortical spindles, and hippocampal sharp-wave ripples playing particularly important roles [42, 80]. While the mechanism linking sleep oscillations to memory performance is not fully understood, it is hypothesized that oscillatory activity enables selective synaptic plasticity by organizing during sleep the reemergence of activity patterns that were present during wake. Sleep spindles have been causally linked to new memory formation in pharmacological studies [50]. In the present study, we do not find disease-altered or age-impacted sleep spindles, which indicates that they are likely not implicated in the known memory deficits found in children with DMD.

Although in our data thalamocortical spindles do not appear to be directly affected by DMD, our characterization of their coordination with SWA showed approximately one-tenth of spindles coupled during N2 while approximately half coupled in N3. This stage difference pattern replicates research by Hahn et al. (2020) in typically developing children and adolescents (age groups approximately 9.5 and 16 years). However, Hahn et al. [81] appeared to find a significantly higher rate of coupling in both children and adolescents than we found in our sample, almost double the rate in N2 and approximately 30% higher in N3. This difference could be the result of the larger time window in their co-occurrence detection. We also did not find an increase in coupling strength across development as they found between children and adolescents. This comparison suggests that despite an overall stage pattern similarity, we are observing impaired coupling, both rate and development, in DMD patients. Prior work has also demonstrated that the alignment of SOs and spindles impacts sleep-dependent memory performance [47, 82]. Patients with DMD have a broad spectrum of cognitive difficulties, including memory impairment. Our observed impairment of phase locking across development suggests that one underlying mechanism could be dysfunctional sleep-dependent memory consolidation. Further, although the temporal coordination appears to be somewhat maintained in our patient population, we do not see developmental changes demonstrated in typically developing youth. Low rates of SO–spindle coupling are hypothesized to underlie poor overnight sleep-dependent memory consolidation in aging [83]. Our identification of abnormal age-associated sleep features and low rates of spindle–SWA concordance points to actionable therapeutic targets.

In line with our prior analysis of these records, several limitations exist with the current study. Due to the rarity of this genetic disorder, our sample size is small. Further, we evaluated clinically required retrospective sleep records conducted at a tertiary hospital to evaluate the presence of sleep disorders [84]. However, across our age groups, we did not observe differences in sleep apnea events. Additionally, within DMD/BMD populations, genetic heterogeneous abnormalities are present. While it is possible that certain genetic variations might impact the spindle features we evaluated, prior investigation into the neurological impacts of DMD has not identified abnormalities in the thalamocortical circuitry [31, 38]. Due to our small sample size, we are underpowered to evaluate specific genetic, medication, or ambulation influences on sleep physiology features; however, as these factors are all common in the DMD population, evaluating their potential differential influence on sleep brain physiology remains a consequential endeavor for future research, requiring tailored prospective patient samples. Lastly, given the large literature showing the causal role spindles play in sleep-dependent cognition, it will be important for future studies to experimentally evaluate if patients with DMD show similar sleep-dependent performance benefits that are correlated to sleep spindles.

At present, there is a limited scope in research studies characterizing sleep spindles in pediatric patient populations. In the few reported case studies involving pediatric patients with disorders that encompass musculature symptoms, we found mixed results. Case studies of young children with cerebral palsy show some have typical sleep patterns while others have altered sleep stage architecture and low spindle density [85]. Case studies of youth with neurodegenerative lysosomal-storage disorders show a loss of spindles that appears to mirror disease-caused cortical atrophy [86–88]. Altogether, our research points to a concerning paucity of research in developmental sleep literature involving youth with rare diseases or complex medical conditions. Global estimates of rare diseases suggest that 263–446 million people are affected worldwide with approximately 69.9% having a pediatric onset and another 18% having disease onset that can be either pediatric or adult [89]. Sleep health is a targetable and feasible intervention that can improve physical health, mood, and cognition [90]. This research (in combination with our previous work) is a foundational demonstration that it is possible to identify actionable sleep targets within a specific rare disease. Moving forward, we argue that there is a strong need to focus on pediatric sleep research in rare disease populations and develop tools to enhance the feasibility for children with complicated medical needs to participate in sleep-based research. We further need to develop sleep health interventions to improve sleep malleable elements, such as slow wave sleep, that are sustainable for families of children with rare conditions and can be deployed across development and in a variety of settings.

## Conclusion

We observed age-associated changes in spindle characteristics in our pediatric DMD patient population that were in line with those found in healthy youth. Our work builds on our prior discovery of a significant age-related decline in SOs, suggesting that SOs, but not spindles, are a candidate intervention target to enhance sleep in patients with DMD throughout childhood. Together, our results provide support that sleep can be used as an accessible biomarker for pediatric populations with pathological development and identify specific sleep features that are actionable targets to enhance sleep, and possibly memory and cognition.

## Data Availability

Data is available upon request.
